# Polyamine stress at high pH in *Escherichia coli *K-12

**DOI:** 10.1186/1471-2180-5-59

**Published:** 2005-10-13

**Authors:** Elizabeth Yohannes, Amy E Thurber, Jessica C Wilks, Daniel P Tate, Joan L Slonczewski

**Affiliations:** 1 Department of Biology, Kenyon College, Gambier, OH 43022

## Abstract

**Background:**

Polyamines such as spermine and spermidine are required for growth of *Escherichia coli*; they interact with nucleic acids, and they bind to ribosomes. Polyamines block porins and decrease membrane permeability, activities that may protect cells in acid. At high concentrations, however, polyamines impair growth. They impair growth more severely at high pH, probably due to their increased uptake as membrane-permeant weak bases. The role of pH is critical in understanding polyamine stress.

**Results:**

The effect of polyamines was tested on survival of *Escherichia coli *K-12 W3110 in extreme acid or base (pH conditions outside the growth range). At pH 2, 10 mM spermine increased survival by 2-fold, and putrescine increased survival by 30%. At pH 9.8, however, *E. coli *survival was decreased 100-fold by 10 mM spermine, putrescine, cadaverine, or spermidine. At pH 8.5, spermine decreased the growth rate substantially, whereas little effect was seen at pH 5.5. Spermidine required ten-fold higher concentrations to impair growth. On proteomic 2-D gels, spermine and spermidine caused differential expression of 31 different proteins. During log-phase growth at pH 7.0, 1 mM spermine induced eight proteins, including PykF, GlpK, SerS, DeaD, OmpC and OmpF. Proteins repressed included acetate-inducible enzymes (YfiD, Pta, Lpd) as well as RapA (HepA), and FabB. At pH 8.5, spermine induced additional proteins: TnaA, OmpA, YrdA and NanA (YhcJ) and also repressed 17 proteins. Four of the proteins that spermine induced (GlpK, OmpA, OmpF, TnaA) and five that were repressed (Lpd, Pta, SucB, TpiA, YfiD) show similar induction or repression, respectively, in base compared to acid. Most of these base stress proteins were also regulated by spermidine, but only at ten-fold higher concentration (10 mM) at high pH (pH 8.5).

**Conclusion:**

Polyamines increase survival in extreme acid, but decrease *E. coli *survival in extreme base. Growth inhibition by spermine and spermidine requires neutral or higher pH. At or above pH 7, spermine and spermidine regulate specific proteins, many of which are known to be regulated by base stress. High pH amplifies polyamine stress; and naturally occurring polyamines may play an important role in base stress.

## Background

Polyamines are required for the normal cell growth of *Escherichia coli*, although their functions are poorly understood [1-3]. Polyamines bind nucleic acids and ribosomes, where they are needed for optimal function [4,5]. Excessive intracellular concentrations, however, retard protein synthesis and cell growth [6]. Polyamine metabolism is stimulated by a variety of environmental stresses such as heat shock [7].

The major polyamine of bacteria, putrescine [NH_2_(CH_2_)_4_NH_2_], is synthesized by biosynthetic decarboxylation of arginine and/or ornithine [8,9]. Putrescine is metabolized to spermidine [NH_2_(CH_2_)_3_NH(CH_2_)_4_NH_2_]. Spermine [NH_2_(CH_2_)_3_NH(CH_2_)_4_NH(CH_2_)_3_NH_2_], a longer polyamine commonly produced by eukaryotes, is not produced by *E. coli*. Nevertheless, uptake of exogenous spermine fullfills the bacterial requirement for polyamines [4,5]. Spermine and spermidine do not undergo catabolism by *E. coli*, although excess concentrations are acetylated by polyamine acetyltransferase [10,11].

In the human colon, bacteria excrete putrescine and cadaverine during digestion of high-protein foods. Exposure of colonic epithelium to these polyamines stimulates human cell proliferation and leads to colonic tumors [12], which can be treated by drugs that deplete polyamine content [13]. Thus the modulation of polyamine metabolism under conditions of the gut is an important medical concern.

The inhibition of growth by excess polyamines is amplified at high pH [14]. Polyamine stress enhances translation of the growth phase sigma RpoS [15], whose role in stationary-phase survival involves high pH [16]. A possible explanation for the amplification of polyamine stress at high pH is that polyamines become deprotonated and neutralized, thus capable of crossing the cell membane as membrane-permeant weak bases. Base-dependent uptake could augment the uptake through transporters [17]. The uptake of amines leads to their accumulation proportional to the transmembrane pH difference (ten-fold for each pH unit). Only a small fraction of an amine needs to be unprotonated (less than 1%) in order for significant membrane passage to occur. The pK_a _values of linear polyamines range from pK_a _= 8.3 to 11.6; for example, Ref [18] reports for 30 mM spermidine values of pK_a1 _= 8.6, pK_a2 _= 10.0, pK_a3 _= 11.1. However, literature reports vary for different conditions, and polyamine protonation levels under biological conditions are further influenced by complexing with fatty acids and phospholipids.

At low pH, on the other hand, the transmembrane pH difference (ΔpH) limits the entry of exogenous amines; and cytoplasmic production of membrane-permeant amines can enhance bacterial growth. A pH-dependent source of amines in *E. coli *is the degradative arginine and ornithine decarboxylases (AdiA and SpeF respectively) and lysine decarboxylase (CadA), which are induced anaerobically at low pH [19]. The generation of putrescine (by AdiA) or cadaverine (by CadA), followed by excretion via cotranscribed transporters, neutralizes the acidic external environment [20]; for review, see [21,22].

At low-to-neutral pH, *E. coli *polyamines block the porins OmpF and OmpC, decreasing membrane permeability [23-25]. It was proposed that polyamines contribute to *E. coli *survival in extreme acid, below the growth range [24]; a phenomenon termed acid resistance or acid survival [21,22]. An OmpC mutant in which the porin fails to be blocked by cadaverine shows decreased survival at low pH (acid resistance) in the presence of cadaverine [24]. To our knowledge, it has not been shown directly that exogenous polyamines enhance acid resistance of wild-type cells. If polyamines do enhance survival in extreme acid, they could assist *E. coli *and other enteric pathogens during their passage through the stomach [26].

The interactions between pH and polyamines however remain poorly characterized, and are often discounted in studies of polyamine stress. For example, a recent major study of polyamine-mediated gene regulation does not address pH [3]. We report the effect of exogenous spermine and other polyamines on *E. coli *survival in extreme acid or extreme base. We also present protein profiles of *E. coli *exposed to exogenous polyamines under neutral and alkaline pH conditions, in the context of known pH-dependent expression profiles [27,28].

## Results

### Survival at extreme pH

*E. coli *grown at moderate pH values possess mechanisms of protection against more extreme pH; these mechanisms are typically induced during stationary phase, or during growth near the acid or base end of their pH range [21,22]. We tested the effects of spermine, putrescine, spermidine and cadaverine on survival rates in extreme acid or base, outside the range of pH permitting growth. Stationary-phase cultures of *E. coli *K-12 were diluted 1000-fold in LBK pH 2.0, or in LBK 100 mM CAPS pH 9.8, and incubated for 2 h at 37°C, then dilutions were plated on LBK agar. The pH of the incubation medium was tested before and after culture inoculation, and no change in pH was seen. Under these conditions of extreme acid or base, overall survival (based on LBK plate count) was about 20% compared to that of a control culture diluted in LBK.

**Figure 1:**
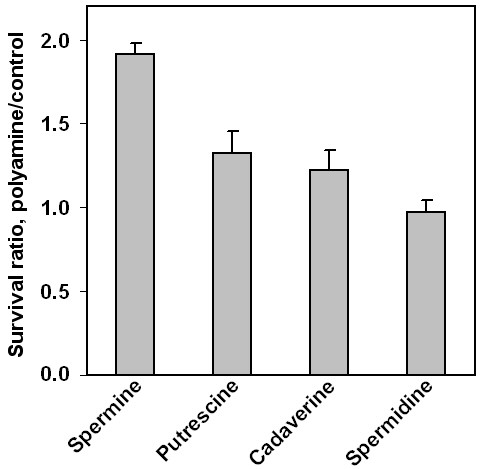
**Extreme-acid survival in the presence of polyamines. **The mean survival at pH 2 after 2 h in the presence of each polyamine (10 mM) was divided by the mean survival of a sample diluted from the same overnight culture without exogenous polyamines. The mean of six independent cultures is shown, +/- SEM.

At pH 2.0, the presence of 10 mM spermine doubled the proportion of *E. coli *survivors (Figure 1). Putrescine and cadaverine increased survival by smaller increments, and spermidine had no effect. At pH 9.8, however, all four polyamines tested decreased survival by more than 100-fold. Thus, the presence of polyamines enhanced survival in acid but drastically diminished survival in extreme base.

### Growth rates with spermine or spermidine

In order to observe polyamine stress and protein expression during growth, we treated cultures with exogenous spermine and spermidine. These polyamines were selected because they do not undergo the rapid catabolism that occurs to putrescine, the major endogenous polyamine of *E. coli*. Spermine interacts with cell components in similar ways as other polyamines found in *E. coli *[5], and it effectively enters cells [17].

**Figure 2:**
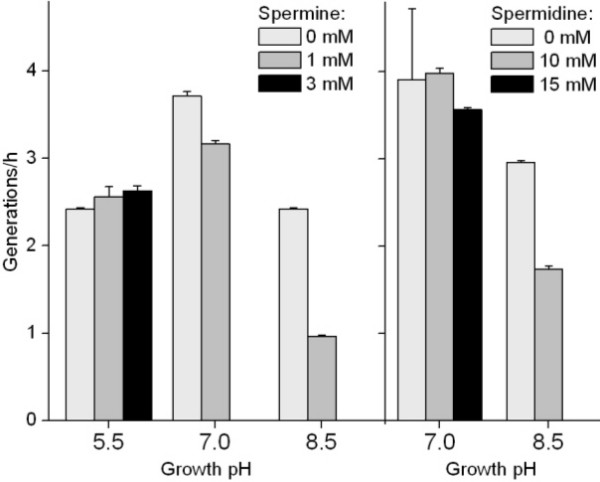
**Generation time with polyamines, as a function of pH. ***E. coli *was grown aerobically at 37°C in LBK buffered at pH 5.5, 7.0, or 8.5, with or without 1 mM spermine or 10 mM spermidine. Overnight cultures were diluted 1000-fold, and growth rates were measured between OD_600 _values of 0.1 and 0.2. Error bars represent SEM (N = 3).

Growth rates were observed in the presence and absence of spermine at pH 5.5, 7.0, or 8.5 (Figure 2). At pH 7.0 or higher, 3 mM spermine eliminated growth, and at pH 8.5 as little as 1 mM spermine substantially decreased growth. At pH 5.5, however, spermine had no effect up to concentrations as high as 10 mM, and spermidine had no effect at 20 mM (data not shown). Spermidine required larger concentrations to affect growth at high pH. At pH 7.0, spermidine concentrations up to 15 mM did not affect the generation time, but at pH 8.5, 15 mM spermidine prevented growth. Thus both spermine and spermidine showed base-dependent depression of E. coli growth, although spermidine required higher concentrations.

### 2-D protein gels

The protein profiles of *E. coli *in the presence and absence of exogenous spermine or spermidine were observed at pH 7.0 and at pH 8.5, using methods that previously revealed pH-dependent protein profiles [28,29]. The concentrations of each polyamine were chosen based on Figure 2 so as to cause significant stress at high pH without preventing growth.

**Figure 3:**
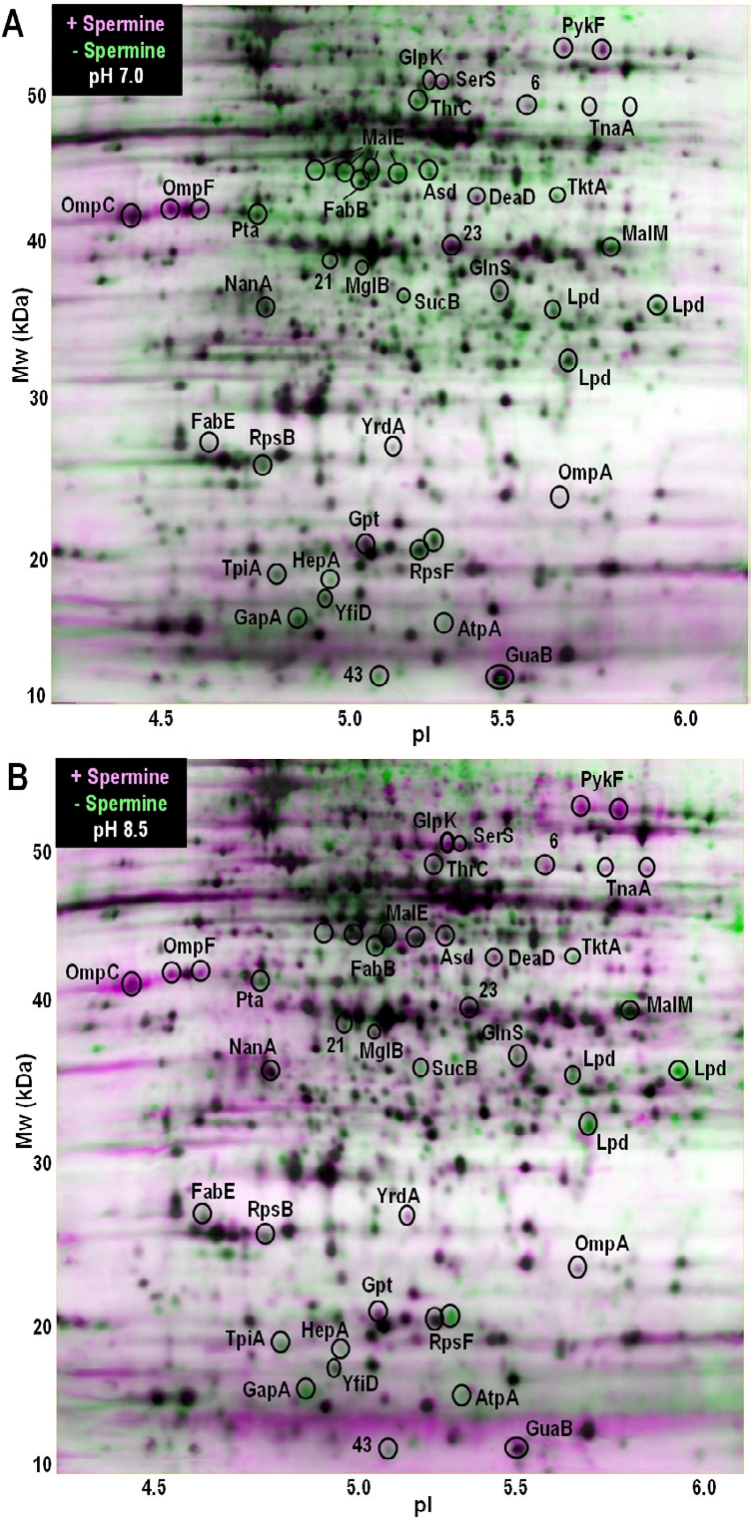
**Protein profiles in the presence or absence of 1 mM spermine. **The horizontal axis represents the approximate pH range of the isoelectric focusing first dimension, and the vertical axis represents the molecular weight (Mw). The "layered view" superimposes two composite images, each composed from three 2-D gels of independently grown replicate cultures. All *E. coli *cultures were diluted 500-fold and grown at 37°C to an OD_600 _of 0.2 in LBK with (pink) or without (green) 1 mM spermine. Culture media were buffered as described under Methods. **(A) **100 mM MOPS, pH 7.0. **(B) **100 mM TAPS, pH 8.5.

**Figure 4:**
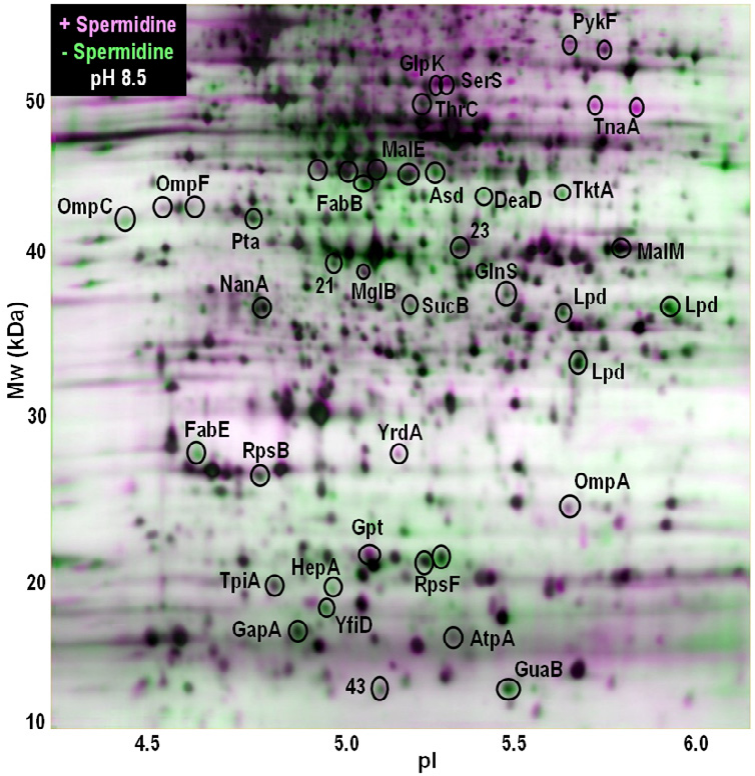
**Protein profiles in the presence or absence of 10 mM spermidine. **Cultures were grown as for Fig. 2B in LBK 100 mM TAPS, pH 8.5, with (pink) or without (green) 10 mM spermidine.

The composite 2-D protein profiles are shown in Figures 3 and 4, and the results of quantitative analysis of pairwise comparisons are shown in Table 1. At pH 7.0, 1 mM spermine increased the expression of eight proteins, including PykF, GlpK, SerS, DeaD, OmpC and OmpF. On the other hand, 19 proteins were repressed, including acetate-inducible proteins (YfiD, Pta, Lpd), the RNA polymerase-binding protein RapA (HepA), and the fatty acid synthesis proteins FabB, FabE. At pH 8.5, spermine induced all the proteins induced at pH 7.0, plus four additional proteins: TnaA, OmpA, NanA, and YrdA. Spermidine regulated most of the same proteins as spermine, although 10-fold higher concentration was required.

The proteins induced by spermine and spermidine include known base-inducible proteins such as TnaA, which deaminates several amino acids [29,30], as well as base-inducible GlpK, OmpA, and OmpF [27]. Proteins inducible at low pH or by acetate, however, were repressed by spermine, including Lpd, Pta, SucB, TpiA, and YfiD [27,31]. By contrast, at pH 5.5, spermine and spermidine had no effect on protein profiles (data not shown). The lack of effect of polyamines at low pH is consistent with their exclusion from the cell as permeant bases, in equilibrium with the trans-membrane ΔpH.

Several porins (OmpC, OmpF, and OmpA) showed increased expression in the presence of spermine. While blockage of porin function by polyamines is eliminated at pH 9.5 [32], some porin blockage may occur as high as pH 8.5. A possible interpretation could be that cells respond to porin blockage by overexpressing the porins.

The polyamines consistently repressed RapA, a general transcriptional activator that stimulates recycling of RNA polymerase [33]; its expression is growth phase dependent, peaking in early log phase [34]. The repression of RapA could contribute to the growth inhibition by polyamines at high pH.

## Discussion

We show that the presence of spermine enhances bacterial survival in extreme acid, as well as diminishing survival in extreme base. This finding is of medical significance, as it suggests that exogenous polyamines enhance survival during passage through the stomach to the colon. The lumen of the colon, at pH 6–8, normally contains millimolar concentrations of polyamines [12], and levels may reach 5–10 mM in children with diseases of nutrient malabsorption such as cystic fibrosis [35]. Polyamines are implicated in colonic hyperproliferation and cell migration, leading to tumorigenesis.

The polyamine enhancement of extreme-acid survival could involve several possible mechanisms. Amines produced by amino-acid decarboxylases can neutralize acidity, a factor in acid resistance [19]. The OmpC porin is blocked by the polyamine cadaverine at low to neutral pH; the blockage of porins could protect cells from acidification [23-25]. Polyamines could also protect the cell's DNA and RNA from effects of internal acidification during extreme-acid exposure [36].

In protein profiles, spermine and spermidine showed base-dependent regulation of 31 proteins, including nine proteins known to respond to base stress. Polyamines have numerous effects upon cell function; their effects could be enhanced at high pH by ΔpH-driven uptake of polyamines. Alternatively, polyamines could amplify base stress by taking up protons within the cytoplasm, thus requiring the cell to spend more energy maintaining pH homeostasis.

Our results of protein expression differ significantly from the report of putrescine-dependent genes by Yoshida and colleagues [3]. For example, Ref. [3] reports putrescine induction of acid stress genes such as *dps*, *hdeA*, *hdeB*, *gatABD*, and the flagellar regulon. We however find mainly induction of base stress proteins and repression of acid stress proteins. The difference may relate to the fact that in Ref. [3], the growth medium used was glucose-minimal Medium A, with pH unspecified but presumably buffered with phosphate at pH 7, with cells grown to late log phase. These growth conditions involve substantial acidification and acetate production. Furthermore, the putrescine microarray study was performed using a mutant deficient for putrescine conversion to spermidine, which requires exogenous polyamines for optimal growth. The effects of acidification could have been amplified by the growth defect of the polyamine-deficient strain; thus, culture acidification could explain why Ref. [3] found acid stress induction. Our proteomic experiments, however, used an *E. coli *K-12 strain with no known polyamine defects, and growth pH was maintained by buffering at pH 7.0 or 8.5.

## Conclusion

It has been shown that polyamines increase survival in extreme acid, but decrease *E. coli *survival in extreme base. At or above pH 7, spermine and spermidine induce specific proteins, including those associated with base stress. Base stress and base-enhanced polyamine uptake should be considered during all studies of polyamine stress.

## Methods

### Growth conditions

*E. coli *K-12 strain W3110 was grown overnight in potassium-modified Luria broth (LBK) (10 g/l of tryptone, 5 g/l of yeast extract, 7.45 g/l of KCl). The overnight cultures were diluted 500-fold into 20 ml of buffered medium supplemented with 1 mM spermine or 10 mM spermidine. The buffers used included 3-(N- morpholino)propanesulfonic acid (MOPS) (pK_a _7.01), 3- [N-tris(hydroxymethyl)methyl]- 3-aminopropanesulfonic acid (TAPS) (pK_a _8.11), and cyclohexyl-3-amino-1-propanesulfonic acid (CAPS) (pK_a _10.4). Buffers and polyamines were purchased from Research Organics and from Sigma. The pH values of media were adjusted by using KOH to avoid extra sodium ions, which stress cells at high pH [37]. The pH was tested after culture growth to ensure that the values were maintained at ± 0.1 pH unit of the pH of the original uninoculated medium. For 2-D gels, all cultures were grown aerobically in baffled flasks rotated at 240 cycles/sec at 37°C until the OD_600 _reached 0.2.

### Survival assays

To assay survival in extreme acid, overnight cultures of bacteria in LBK 50 mM MOPS pH 7.0 were diluted 1000-fold in LBK adjusted to pH 2.0. After 2 h incubation at 37°C, the pH of the culture was tested to confirm maintenance at pH 2.0. Cells were plated on LBK agar at 37°C. For survival in extreme base, overnight cultures in LBK 50 mM TAPS pH 8.5 were diluted 1000-fold in LBK 100 mM CAPS pH 9.8, incubated for 2 h at 37°C, and the pH tested to confirm maintenance at pH 9.8. Dilutions were plated on LBK. For either acid or base experiments, survival was normalized to that of control samples diluted in LBK 50 mM MOPS pH 7.0 and plated directly without incubation.

### 2-D protein gel electrophoresis

2-D gel electrophoresis of proteins was performed as described [28,31,38]. The protein mixtures were first separated by isoelectric focusing using 18-cm polyacrylamide gel strips with an immobilized pH 4 to 7 gradient (AP Biotech). The second dimension was an electrophoretic gel slab containing 11.5% acrylamide, and the proteins were silver-stained. Certain stained proteins commonly appear as isoforms in multiple spot positions [28].

Protein spot densities were quantified using the Compugen Z3 v.3.0 software (Compugen, Tel Aviv, Israel). The differential expression ratio (DE) of the spot densities for two growth conditions (experimental and control) was computed by pairwise comparisons of a set of three gels from each of two growth conditions. A protein spot was considered a candidate for significant induction if seven of nine pairwise comparisons produced a DE greater than or equal to 1.5 or less than 0.67. For each protein, the log10 of all nine DE values was computed, and the mean log10 DE (LDE) was considered a measure of induction (positive values) or repression (negative values) (Table 1).

### Protein identification

Proteins having a significant LDE were identified either by MALDI-TOF analysis at the Proteomic Mass Spectrometry Laboratory at the University of Massachusetts [39] or by positional comparison with previous gels in which proteins had been identified by MALDI-TOF analysis or N-terminal sequencing [28,31]. For database searches of MALDI-TOF masses the Protein Prospector site was used [40] at the University of California at San Francisco.

## Authors' contributions

EY designed the study, conducted the 2-D gels, and drafted the manuscript. AT and JW designed and conducted the survival assays. DPT conducted growth curves. JLS conceived of the study, contributed to its design, and revised the manuscript.

## Figures and Tables

**Table 1:** Proteins showing differential expression on 2-D gels.

		**Differential expression**^1^			
			
**Spot no.**	**Protein**	**Spermine (1 mM)**		**Spermidine (10 mM) pH 8.5**	**Known or predicted function**
		**pH 7.0**	**pH 8.5**		
1	PykF	0.34 ± 0.09	(+)	(+)	Pyruvate kinase
2	PykF	0.39 ± 0.06	(+)	(+)	Pyruvate kinase
3	GlpK	0.68 ± 0.09	0.63 ± 0.12	0.50 ± 0.09	Glycerol kinase
4	SerS	0.83± 0.08	0.60 ± 0.1	0.60 ± 0.1	Serine-tRNA ligase
5	ThrC	-0.34 ± 0.06			Threonine synthase
6		0.51 ± 0.03	0.81 ± 0.07		
7	TnaA		0.92 ± 0.06	0.87 ± 0.09	Tryptophanase
8	TnaA		0.53 ± 0.07	0.77 ± 0.07	Tryptophanase
9	MalE	-1.04 ± 0.05			Maltose-binding, periplasmic
10	MalE	-1.0 ± 0.00			Maltose-binding, periplasmic
11	MalE				Maltose-binding, periplasmic
12	MalE	-0.74 ± 0.08			Maltose-binding, periplasmic
13	Asd	-0.54 ± 0.10		-0.42 ± 0.07	Aspartate semialdehyde dehydrogenase
14	FabB	(-)	(-)	-0.64 ± 0.11	Beta-Ketoacyl-ACP synthase I
15	DeaD	0.27 ± 0.03	0.41 ± 0.08		ATP-dependent RNA helicase
16	TktA	-0.46 ± 0.11	(-)	-0.48 ± 0.07	Transketolase
17	OmpC	(+)	0.56 ± 0.05		Outer membrane protein C
18	OmpF	0.96 ± 0.03	0.74 ± 0.15		Outer membrane porin
19	OmpF	0.56 ± 0.05	0.90 ± 0.08		Outer membrane porin
20	Pta	(-)	-0.50 ± 0.15	(-)	Phosphate aceyltransferase
21		-0.47 ± 0.06	(-)	(-)	
22	MglB			0.23 ± 0.02	Galactose-binding protein
23		0.69 ± 0.11	(+)		
24	MalM	-59 ± 0.10			Maltose periplasmic protein
25	SucB	-0.25 ± 0.02	-0.89 ± 0.08		Dihydrolipoamide Succinyltransferase
26	GlnS	(-)	-0.59 ± 0.11	-0.60 ± 0.1	Glutaminyl-tRNA synthetase
27	NanA		0.49 ± 0.12		Putative N-acetylmanosamine-6-phosphate 2-epimerase
28	Lpd	(-)	-0.79 ± 0.1	-0.69 ± 0.1	Dihydrolipoamide dehydrogenase
29	Lpd	(-)	-0.55 ± 0.1	-0.72 ± 0.17	Dihydrolipoamide dehydrogenase
30	Lpd	(-)	-0.58 ± 0.1	-0.27 ± 0.05	Dihydrolipoamide dehydrogenase
31	FabE		-0.24 ± 0.04	-0.82 ± 0.07	Acetyl-CoA carboxylase
32	RpsB	(-)	-0.26 ± 0.05		30S ribosomal subunit protein S2
33	YrdA		0.26 ± 0.04	0.44 ± 0.12	
34	OmpA		0.27 ± 0.04	0.43 ± 0.09	Outer membrane protein A
35	RpsF	-0.22 ± 0.01	-0.85 ± 0.09	-0.50 ± 0.12	30S ribosomal subunit protein S6
36	RpsF	-0.22 ± 0.01			30S ribosomal subunit protein S6
37	Gpt	0.18 ± 0.03	0.40 ± 0.08	0.58 ± 0.1	
38	TpiA	-0.28 ± 0.03	-0.57 ± 0.06	(-)	Triosephosphate isomerase
39	RapA (HepA)	-0.61 ± 0.02	-0.76 ± 0.09	-0.63 ± 0.11	RNA polymerase binding protein
40	YfiD	-0.27 ± 0.02	-0.21± 0.02	-0.37 ± 0.03	Pyruvate formate-lyase homolog
41	GapA	-0.51 ± 0.1	-0.87 ± 0.06	-0.50 ± 0.13	Glyceraldehyde 3-phosphate dehydrogenase A
42	AtpA	-0.27 ± 0.07			ATP synthase subunit alpha
43		-0.74 ± 0.02	(-)	(-)	
44	GuaB			-0.75 ± 0.1	Inosine-5'-monophosphate dehydrogenase

^1^Relative differential expression of protein, shown as LDE ± standard error (n = 9), was determined as described under Materials and Methods. Symbols: (+), induced; (-) repressed, representing spots that showed differential expression based on visual inspection, although not quantifiable by the Z3 software.
